# Metabolic Disorders: A Case Report of the Dark Side of Ammonia Inhalation Poisoning

**DOI:** 10.7759/cureus.108399

**Published:** 2026-05-06

**Authors:** María Rodil Riera, Pablo Lozano Cuesta, Liliana Pérez Martinez, María Rodriguez Pericacho, Marta Íscar Urrutia

**Affiliations:** 1 Pulmonology, Hospital Universitario de Cabueñes, Gijón, ESP; 2 Pulmonology, Hospital Universitario Central de Asturias, Oviedo, ESP

**Keywords:** ammonia (nh3+), bronchospasm, metabolic acidosis, poisoning, respiratory failure.

## Abstract

This report describes a case of metabolic acidosis secondary to acute ammonia (NH_3_^+^) inhalation, a complication rarely documented in medical literature. The patient, a 73-year-old man, developed dyspnea after inhaling NH_3_^+^ while cleaning a bathroom. Initial diagnoses included acute kidney injury(AKI) and global respiratory failure with mixed acidosis secondary to NH_3_^+^ inhalation. Although the patient showed initial respiratory improvement, he experienced moderate renal function deterioration, hyperkalemia, and metabolic acidosis, which required bicarbonate treatment. After 24 hours, his clinical and analytical parameters improved, allowing for hospital discharge.

This case highlights the need to consider metabolic alterations in patients exposed to acute NH_3_^+^ inhalation and underscores the importance of a multidisciplinary approach to managing respiratory and metabolic complications, thus contributing valuable insights to the literature on ammonia intoxication management and treatment.

## Introduction

Ammonia (NH₃) is a colorless, water-soluble chemical compound. Ammonia poisoning may occur in occupational settings, the agricultural and industrial sectors, or as a result of domestic accidents involving cleaning products [1]. When NH₃ contacts water in the tissues, it is converted to ammonium hydroxide (NH₄OH) in an exothermic reaction that causes chemical injury and liquefactive necrosis. Acute exposure can lead to irritation and burns of the skin and mucous membranes and can compromise the respiratory (burns, edema, and/or airway obstruction) and digestive systems. At high concentrations, it may affect consciousness and cause cardiovascular complications [1,2].

The metabolic impact associated with ammonia inhalation is not well described in the literature. Ammonia inhalation may lead to a series of disturbances whose severity depends on the duration and concentration of exposure [3]. Reported alterations include acid-base disorders, electrolyte imbalances, and, in some cases, acute kidney injury (AKI), although the underlying mechanisms remain incompletely understood. We present a case of a patient with AKI, acute respiratory failure, electrolyte imbalances, and metabolic acidosis in the context of acute ammonia inhalation.

## Case presentation

The patient, a 73-year-old retired plumber, presented to the Emergency Department with dyspnea after using a domestic liquid cleaning product containing ammonia (NH₃) and inhaling its vapor while showering for approximately 15 minutes. The exact concentration of exposure was unknown. A former smoker (15 years since cessation; cumulative 120 pack-years) and heavy alcohol user (three units/day, previously more than 10 units/day), his medical history included hypertension, hyperuricemia, grade II/IV aortic insufficiency, and chronic obstructive pulmonary disease (Global Initiative for Chronic Obstructive Lung Disease (GOLD) stage 3A). There was no known history of chronic kidney disease or chronic liver disease. The patient experienced progressive worsening of baseline dyspnea (previously mMRC 2) until it became disabling (mMRC 4) over a few hours and reported wheezing. Physical examination revealed a respiratory rate of 30 breaths/min, accessory muscle use, oxygen saturation of 85%, and facial flushing, with no cyanosis or hypotension documented. Auscultation indicated decreased breath sounds with wheezing. Supplemental oxygen was administered.

Blood analysis revealed AKI (creatinine 1.57 mg/dL) and electrolyte imbalances, including hyperkalemia (5.5 mmol/L), hypocalcemia (2.14 mmol/L), hypophosphatemia (0.78 mmol/L), and hypomagnesemia (0.62 mmol/L). Arterial blood gas analysis showed global respiratory failure with mixed acidosis (pH 7.28, pO₂ 52 mmHg, pCO₂ 53 mmHg, bicarbonate (HCO₃⁻) 21.8 mmol/L, base excess (BE) -2 mmol/L), with a normal anion gap. Chest X-ray and ECG showed no significant abnormalities.

The initial treatment included nebulized bronchodilators (three doses of ipratropium 500 mcg, salbutamol 2.5 mg, and a single dose of budesonide 1 mg) and systemic corticosteroids (40 mg methylprednisolone IV). Auscultation improved, with resolution of bronchospasm and normalization of pCO₂. However, metabolic acidosis persisted, with declining HCO₃⁻ and BE (pH 7.30, pO₂ 58 mmHg, pCO₂ 44 mmHg, HCO₃⁻ 20.1 mmol/L, BE -5.1 mmol/L).

Due to an uncertain prognosis, the patient was admitted for monitoring, and fluid therapy was initiated. No clear alternative precipitants of AKI (such as hypotension or nephrotoxic drugs) were identified. Within hours, he developed worsening hyperkalemia (K⁺ 6.9 mmol/L) despite improved renal function (creatinine 0.99 mg/dL), with persistent metabolic acidosis, with blood lactate levels within normal range, prompting the initiation of oral bicarbonate. ECG showed no significant abnormalities and no clinical signs of hyperkalemia were observed. Despite severe hyperkalemia, the absence of electrocardiographic changes and clinical manifestations allowed a conservative approach, prioritizing correction of metabolic acidosis with close monitoring, which led to progressive normalization of potassium levels. Specific electrolyte replacement was not required.

After 24 hours, clinical and analytical improvements were observed, with normalization of bicarbonate (HCO₃⁻ 24 mmol/L) and potassium (K⁺ 5.1 mmol/L). Arterial blood gas parameters normalized within 24 hours. The patient was discharged after 48 hours of hospitalization. Urine studies, lactate levels, and blood ammonia levels were not obtained and were not available for analysis. Laboratory findings are summarized in Tables 1, 2.

**Table 1:** Laboratory findings (biochemistry)

Parameter	Admission	Worst value	24-48 h	Normal range
Creatinine (mg/dL)	1.57	1,57	0.99	0.7-1.3
Potassium (mmol/L)	5.5	6.9	5.1	3.5-5.0
Calcium (mmol/L)	2.14	2,14	-	2.15-2.55
Phosphate (mmol/L)	0.78	0,78	-	0.80-1.50
Magnesium (mmol/L)	0.62	0,62	-	0.70-1.00
Sodium (mmol/L)	Within normal range		Within normal range	135-145

**Table 2:** Arterial blood gas analysis

Parameter	Admission	After treatment	24 h	Normal range
pH	7.28	7.30	Within normal range	7.35-7.45
pO₂ (mmHg)	52	58	Within normal range	75-100
pCO₂ (mmHg)	53	44	Within normal range	35-45
HCO₃⁻ (mmol/L)	21.8	20.1	24	22-26
Base excess (mmol/L)	-2	-5.1	Within normal range	-2 to +2
Anion gap	Within normal range	Within normal range	Within normal range	8-16

At a two-month follow-up, the patient had returned to his previous baseline with grade 2 modified Medical Research Council (mMRC) dyspnea, with no evidence of further intoxication episodes.

## Discussion

This case illustrates AKI, global respiratory failure, and metabolic acidosis in the context of ammonia inhalation. Respiratory failure was likely related to bronchospasm and airway inflammation, which responded to bronchodilators and corticosteroids [2,4]. Experimental studies suggest that adenosine triphosphate (ATP) release may mediate airway inflammation after ammonia exposure, contributing to pulmonary dysfunction [5].

Metabolic alterations associated with ammonia inhalation remain incompletely understood. In this case, the patient initially presented with mixed acidosis, which later evolved into predominantly metabolic acidosis with a normal anion gap. The proposed mechanisms include bicarbonate consumption following ammonium formation and impaired renal acid excretion. In addition, ammonia may interfere with cellular metabolism and mitochondrial function, promoting anaerobic pathways and acidemia [3,6].

Electrolyte disturbances were also observed. Hyperkalemia was likely multifactorial, related to AKI and metabolic acidosis. In contrast, hypocalcemia, hypophosphatemia, and hypomagnesemia could not be directly attributed to ammonia exposure, and alternative mechanisms should be considered.

Renal involvement in ammonia toxicity is not well characterized. Experimental and clinical data suggest that ammonia may induce renal injury through oxidative stress, tubular toxicity, and altered renal hemodynamics, although evidence in humans remains limited [6,7]. In this case, AKI occurred without hypotension or other clear precipitating factors, suggesting a possible association with ammonia exposure, although causality cannot be definitively established.

Management is mainly supportive and includes oxygen therapy, bronchodilators, corticosteroids, and fluid resuscitation. Bicarbonate therapy may be considered in cases of significant metabolic acidosis with hyperkalemia [4,8]. In our patient, correction of acidosis was associated with normalization of potassium levels.

This report has several limitations. Urine studies and renal imaging were not performed, limiting full characterization of AKI. In addition, lactate and blood ammonia levels were not measured, and the exact concentration of exposure was unknown. These factors restrict causal interpretation and highlight the need for cautious interpretation of the findings.

Overall, this case highlights the importance of recognizing metabolic and renal alterations in patients exposed to ammonia inhalation and supports a comprehensive clinical and biochemical assessment in these situations.

## Conclusions

This case underscores the importance of recognizing metabolic disturbances following ammonia inhalation, particularly electrolyte imbalances and acid-base disorders that may evolve despite initial respiratory improvement. It highlights the need for close monitoring and a multidisciplinary approach to manage both respiratory and metabolic complications effectively. Further research is warranted to better understand the underlying mechanisms of these alterations and to optimize management strategies.
